# Biomarkers in Liver Disease: Emerging Methods and Potential Applications

**DOI:** 10.1155/2012/437508

**Published:** 2012-11-07

**Authors:** Guruprasad P. Aithal, Neil Guha, Jonathan Fallowfield, Laurent Castera, Andrew P. Jackson

**Affiliations:** ^1^NIHR Biomedical Research Unit in Gastrointestinal and Liver Diseases at Nottingham University Hospitals NHS Trust and the University of Nottingham, Nottingham NG7 2UH, UK; ^2^MRC/Centre for Inflammation Research, QMRI, University of Edinburgh, Edinburgh EH16 4TJ, UK; ^3^Department of Hepatology, CRB3 INSERM U 773, Hôpital Beaujon, Assistance Publique Hôpitaux de Paris, Université Paris-7, 75205 Paris Cedex, France

## 1. Introduction

Biomarker research represents an evolving area within hepatology. The growing burden of global liver disease, the absence of symptoms until late in the natural history of a disease which may take decades to manifest, the presence of an invasive reference test (liver biopsy) to assess disease severity, and the lack of robust tools to assess the efficacy of therapeutic interventions are some of the key drivers for this research.

The National Institute of Health defines a biomarker as “A characteristic that is objectively measured and evaluated as an indicator of normal biologic processes, pathogenic processes, or pharmacologic responses to a therapeutic intervention” [1]. Moreover, biomarkers can be classified into hierarchical systems based on their ability to assess natural history (type 0: prognosis), biological activity (type 1: response to therapy), and therapeutic efficacy (type 2: surrogate for clinical efficacy) [2].

The spectrum of pathological injury that occurs in liver disease including steatosis, necroinflammation, apoptosis, and fibrosis enhances the pool of potential biomarkers. Furthermore, advances in technology platforms have created an exponential rise in the discovery of putative mediators of pathophysiological injury. This has been countered by the growing need to align surrogate markers of injury with clinical consequences of injury in order to achieve diagnostic, prognostic, and therapeutic effectiveness. This timely special edition comprises original articles and reviews in the subject areas of biomarker discovery, biomarkers of liver injury, and biomarkers to assess the consequences of liver injury. 

## 2. Methods of Biomarker Discovery 

Advances in instrumentation design have driven biomarker discovery. The advent of modern biological mass spectroscopic techniques in the 1990s and the evolution of 2-dimensional polyacrylamide gel electrophoresis (2D SDS PAGE) from a highly specialist technique to one that could be carried out in most laboratories around the world drove the development of large-scale ‘omics biomarker discovery projects. Advances in microlitre flow rate HPLC, that could be coupled directly to mass spectrometers (nano-LC/MS), and computing to analyse the data gave further impetus to this work. It became possible to quantify and identify many thousands of proteins from diseased and healthy tissue in a single experiment. Biomarker discovery projects ([3] metabonomics; [4] lipidomics; [5] proteomics; [6] SELDI and transcriptomics) demonstrate the ability to identify novel markers of liver disease. Proteomics, transcriptomics, lipidomics, and metabonomics offer the ability to discover completely novel markers of disease and its progression. This *de novo* approach to biomarker discovery leads to a great challenge of marker validation. There may be little or no obvious mechanistic connection between the putative marker and disease, demonstrating that a link can be very time and resource intensive.

Mechanism-focussed biomarker discovery has also benefited from advances in instrumental design and technology. These projects are based on prior disease knowledge and are much more limited in scope but, if successful, are more likely to identify a disease-relevant marker. Standard ELISA assays methodologies have been developed to use valuable patient samples more efficiently by allowing many analytes to be quantified simultaneously. In array or planar assays, a series of primary antibodies are bound to a surface in discrete spots, sample, and secondary antibody, and detection reagents are passed over the array and the location of the signal is determined using imaging technology. Bead-based technologies rely on a mixture of antibody-labelled beads which are then quantified using flow cytometers or dedicated analysers. From 30 to 50 proteins can be analysed per experiment using panels of antibodies that have been optimised to minimise cross-reactivity. Miniaturisation of liquid handling and high-density microplates, currently up to 1536 samples per plate, reduces reagent and patient sample usage when carrying out enzyme activity-based biomarker discovery. A typical 96-well microplate will require 100 *μ*L reaction mix per well, the high density; 1536-well plates require only 5 *μ*L per well, a reduction of 20-fold in sample consumption. Unfortunately, the additional costs that are incurred to ensure accurate reagent dispensing and reaction monitoring are not trivial. S. K. Hartwell, in this issue, describes an alternative approach using flow injection to minimise reagent consumption where sample numbers and volumes may be limited. The use of commonly available laboratory equipment aims to minimise costs and to open up the technology to laboratories with limited resources.

## 3. Biomarkers of Liver Injury

 The pathological processes of steatosis, necroinflammation, oxidative stress, apoptosis, and fibrosis are common to a number of diverse liver diseases. The ability to define these individual entities is advantageous for determining the mechanistic evidence of efficacy, using biomarkers, for proposed treatment strategies. A difficulty remains that the pathological processes are often interdependent or cocorrelated, and thus, delineating biomarkers specific to one mode of injury can be challenging. This is illustrated by the article in this special edition by N. Mousa and coworkers describing the association of alpha fetoprotein (AFP) and liver steatosis in genotype 4 infection in chronic viral hepatitis. The authors postulate that the elevation in AFP is secondary to increased production from hepatic progenitor cells as a response to regeneration following injury. In this study, steatosis was also associated with the presence of necroinflammation and fibrosis, and thus, it is not clear whether it is the extent of liver injury or steatosis *per se* that leads to the elevation in AFP. There exists a wider debate in the literature on whether benign steatosis (in the absence of significant steatohepatitis or fibrosis) has clinical significance. In viral hepatitis, steatosis is most commonly seen in genotype 3 infection and improves following successful viral eradication [7]. In long-term studies based on pathological features at baseline biopsy, steatosis has not been shown to adversely affect outcome in nonalcoholic fatty liver disease [8, 9]. 

Natural history studies have shown that the presence and stage of fibrosis at the index liver biopsy provide prognostic information about the subsequent rate of fibrosis progression ([10–12] and the development of liver-related outcomes [9, 13]). It is therefore no surprise that over the last decade much of the focus has been to define novel biomarkers based on the pathological presence of fibrosis. The success and limitations of this strategy have been outlined elsewhere [14]. Defining surrogates of pathological entities other than liver fibrosis is both necessary and advantageous for a number of reasons. Liver fibrosis is essentially a generic wound-healing response and final common pathway resulting from a spectrum of hepatic insults. Moreover, particular characteristics of the hepatic scar including the composition and physical/biochemical attributes that limit remodelling and angioarchitectural changes have hitherto made the delivery of effective antifibrotic therapy challenging. The ability to intervene “upstream” in the injury process may yield a larger repertoire of therapies with the allure of enhanced targeting and superior drug profiles. Apoptosis in the liver may be one such example. Whilst the engulfment of apoptotic bodies by activated hepatic stellate cells (HSCs) may induce TGF*β* and collagen-*α*1 synthesis and promote fibrosis, paradoxically, in preclinical models, resolution of fibrosis depends on the removal of activated HSCs via apoptosis. Thus, the detailed characterisation of apoptosis may provide critical insights into both fibrogenesis and fibrinolysis. J. B. Chakraborty and colleagues provide a comprehensive review in this special edition of the mechanisms of apoptosis in the liver, candidate apoptosis-related biomarkers, and the potential for clinical translation (e.g., assessing treatment response and/or monitoring the regression of fibrosis). 

## 4. Biomarkers Assessing the Consequences of Liver Injury 

 Following long-term liver injury, the evolution of liver fibrosis to cirrhosis is associated with (1) architectural disturbance; (2) angiogenesis and haemodynamic changes (intra- and extrahepatic) resulting in portal hypertension; (3) a propensity for carcinogenesis. In the event of the injury not being removed, a proportion of affected individuals will have complications of liver failure, bleeding, hepatocellular carcinoma, and death. The ability of biomarkers (at baseline and/or changing over time) to predict these events directly has the potential to improve prognosis and provide a meaningful assessment of clinical effectiveness (as opposed to therapeutic efficacy indicators such as reduction in fibrosis). In hepatology, the limitations of liver biopsy and rather restrictive pathological scoring systems have encouraged the extrapolation of biomarkers (originally based upon pathological end points) to hard clinical end points. There are a number of studies demonstrating that noninvasive biomarkers (including serum analytes and transient elastography) measured at baseline predict liver-related outcomes between 5 and 8 years [15–17].

In this special edition, original research presented by N. Palaniyappan and colleagues has investigated the prognostic accuracy of validated scoring systems for detecting long-term outcomes in alcoholic hepatitis. These scoring systems showed a uniformly poor prognostic performance in detecting mortality at one year (AUC ranges from 0.5 to 0.66), in contrast to abstinence from alcohol within three to six months of initial diagnosis which was associated with an AUC of 0.83. This not only highlights the importance of abstinence but also that dynamic measurement, in this case of behaviour, can have a significant influence on prognosis in the context of liver disease.

Portal hypertension underpins the major complications of liver disease including variceal bleeding, ascites, and renal failure. Both existing and emerging therapeutic strategies in the context of established cirrhosis are directed towards lowering portal hypertension. The gold standard for its assessment remains the hepatic venous pressure gradient (HVPG). Whilst a wealth of evidence supports its prognostic value and utility in directing management [18, 19], it remains an invasive test that is only available in specialist centres. Thus, the search for robust biomarkers that offer a noninvasive alternative to HVPG is important if portal hypertension is to be assessed in routine clinical practice. The review by V. K. Snowdon and colleagues succinctly outlines the pathophysiological basis of portal hypertension and, in particular, uses examples of recent advances in endothelial cell biology/fibrosis and angiogenesis research to support the rationale for emerging biomarkers in this area.

Hepatocellular carcinoma (HCC) is the fifth leading cause of death from cancer in men, the seventh leading cause of death from cancer in women, and the fastest rising cause of cancer mortality worldwide. The majority of patients present at an advanced stage when treatment options are very limited and, consequently, HCC carries a dismal prognosis (overall median survival of 14 weeks, 1-year survival of 13%). Current screening strategies that rely on AFP and ultrasound are widely accepted but have only modest diagnostic accuracy with sensitivity rates between 25% and 65% [20]. There is an urgent need to discover and implement better diagnostic tools for this malignancy that may permit earlier and more accurate detection and the review by T. Behne and M. S. Copur outlines emerging biomarkers that have potential clinical utility. 

To provide stratified care for patients with liver disease, we urgently need noninvasive tools that can effectively phenotype patients based on their degree of liver injury, natural history, and clinical outcomes. It is unthinkable that the choice of intervention in an individual patient still remains, in many circumstances, an empirical exercise involving “trial and error.” Biomarker research and its dissemination should aim to overcome these barriers to individualising care.

*Guruprasad P. Aithal* *Guruprasad P. Aithal* *Neil Guha* *Neil Guha* *Jonathan Fallowfield* *Jonathan Fallowfield* *Laurent Castera* *Laurent Castera* *Andrew P. Jackson* *Andrew P. Jackson*
